# ALS Along the Axons – Expression of Coding and Noncoding RNA Differs in Axons of ALS models

**DOI:** 10.1038/srep44500

**Published:** 2017-03-16

**Authors:** Nimrod Rotem, Iddo Magen, Ariel Ionescu, Noga Gershoni-Emek, Topaz Altman, Christopher J. Costa, Tal Gradus, Metsada Pasmanik-Chor, Dianna E. Willis, Iddo Z. Ben-Dov, Eran Hornstein, Eran Perlson

**Affiliations:** 1 Department of Physiology and Pharmacology, Sackler Faculty of Medicine, and the Sagol School of Neuroscience, Tel Aviv University, Tel Aviv 69978, Israel; 2 Department of Molecular Genetics, Weizmann Institute of Science, Rehovot, Israel; 3 Brain and Mind Research Institute, Weill Cornell Medicine, Burke Medical Research Institute, White Plains, NY, USA; 4 Bioinformatics Unit, G.S.W. Faculty of Life Sciences, Tel-Aviv University, Israel; 5 Department of Nephrology and Hypertension, Hadassah-Hebrew University Medical Center, Jerusalem, Israel

## Abstract

Amyotrophic lateral sclerosis (ALS) is a multifactorial lethal motor neuron disease with no known treatment. Although the basic mechanism of its degenerative pathogenesis remains poorly understood, a subcellular spatial alteration in RNA metabolism is thought to play a key role. The nature of these RNAs remains elusive, and a comprehensive characterization of the axonal RNAs involved in maintaining neuronal health has yet to be described. Here, using cultured spinal cord (SC) neurons grown using a compartmented platform followed by next-generation sequencing (NGS) technology, we find that RNA expression differs between the somatic and axonal compartments of the neuron, for both mRNA and microRNA (miRNA). Further, the introduction of SOD1^G93A^ and TDP43^A315T^, established ALS-related mutations, changed the subcellular expression and localization of RNAs within the neurons, showing a spatial specificity to either the soma or the axon. Altogether, we provide here the first combined inclusive profile of mRNA and miRNA expression in two ALS models at the subcellular level. These data provide an important resource for studies on the roles of local protein synthesis and axon degeneration in ALS and can serve as a possible target pool for ALS treatment.

The subcellular proteome is closely regulated in order to respond to extrinsic signals and metabolic demands^1^,^2^. In neurons, which are highly polarized cells, localization of mRNAs and miRNAs together with local synthesis events at distinct subcellular compartments, such as the soma, dendrites, axons and synapses, can control the subcellular proteome and thus can facilitate the neurons’ ability to respond to stress^3^,^4^,^5^. Alterations in this process can lead to diverse pathological events in the neurons. Indeed, alterations in subcellular localization of RNA binding proteins was demonstrated in neurodegenerative diseases such as Amyotrophic Lateral Sclerosis (ALS)^6^,^7^,^8^,^9^. This suggests the existence of alterations in ALS subcellular transcriptomes; however, the identity and nature of these RNAs remain unknown. The introduction of next-generation sequencing (NGS) techniques combined with subcellular fractionation enables a wide yet precise view of the subcellular transcriptome, which was previously unattainable.

ALS is a lethal, adult-onset neurodegenerative disease that affects both upper and lower motor neurons^10^. ALS pathology is characterized by neuromuscular junction (NMJ) disruption, axonal degeneration, and neuronal death^11^. One of the mechanisms suggested to initiate ALS cellular pathology is an alteration in RNA metabolism, specifically alterations in subcellular localization of RNA^7^,^10^,^12^,^13^. This hypothesis is reinforced by emerging research implicating diverse aspects of RNA processing in a broad spectrum of neurodegenerative diseases^14^,^15^. Previous research highlighted several related dysfunctions in ALS that further support this notion: axonal transport deficit^16^,^17^,^18^, mutations in RNA binding protein (RBP)^19^,^20^; and dysregulation of mRNA^21^ and microRNA (miRNA)^22^,^23^,^24^. miRNAs participate in the regulation of gene expression in many, possibly all, biological processes, and their dysregulation would be predicted to have broad implications. miRNAs act by binding to their target mRNAs, resulting in translational repression and/or mRNA degradation^25^. As miRNAs can potentially be used as either drugs or drug targets, their identification in gene regulation in both physiological and pathological processes can be of vital medical significance.

Alterations in several metabolic and signaling pathways have been described in ALS. While most cases of ALS are sporadic, several mutated genes have been found to be associated with the disease. Among these are mutations of the superoxide dismutase protein SOD1 and the DNA-binding protein TDP43, perhaps representing two separate disease pathways to ALS^10^.

Here, we focused on RNA dysregulation in a cellular model of motor neuron degeneration. To this end, we optimized a compartmentalized platform in order to segregate motor neuron cell bodies from their axons. Examining two ALS models, SOD1^G93A^ and TDP43^A315T^, and two control models, we isolated all RNA from the axonal and somatic compartments. Extracted RNA was tested to ensure a clean separation of the fractions, and then split and processed in parallel for both mRNA and miRNA profiling using NGS. Sequencing results show an axon-specific transcriptome repertoire that differs from the somatic transcriptome. Analysis of RNA from neurons expressing ALS-related mutations highlights alterations in mRNA and miRNA profiles within both the soma and the axons. Integrated analysis of mRNA and miRNA expression strengthened and highlighted a number of potential drug targets that may be of interest for further research. Overall, our work provides a wide range of data that can be mined for further research in the field of neurodegenerative diseases, particularly ALS.

## Results

### Isolation of RNA from the axonal and somatic compartments

**Figure 1:**
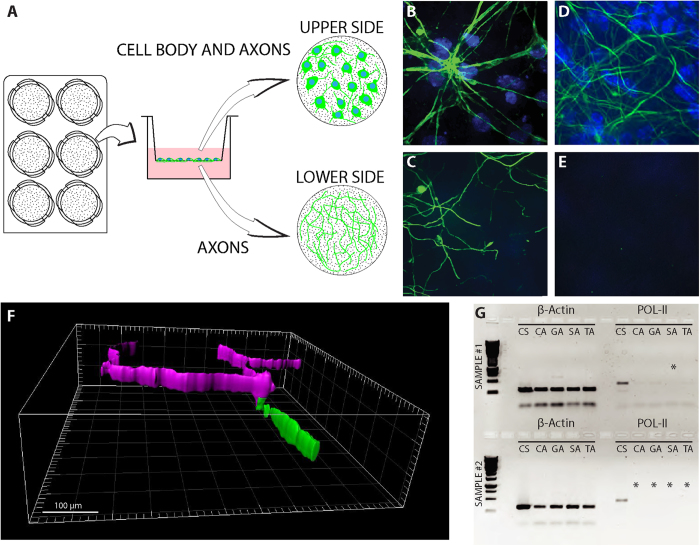
Separation of axons from cell bodies. (**A**) Schematic description of the separation system. Embryonic motor neurons are cultured in a 6-well plate fitted with PET membrane inserts. As neurons grow, axons cross through membrane pores to the lower side of the membrane, allowing the harvest of axons independently of cell bodies, which are located on the upper side. (**B**–**E**) Immunofluorescent staining of neurons on PET membrane: Upper (**B**,**D**) and lower (**C**,**E**) parts of a membrane before (**B**,**C**) and after (**D**,**E**) harvesting axons. Cell nuclei are shown in blue (DAPI). Axons are shown in green (NFH). Scale bar is 20 μm. (**F**) 3D rendering of immunofluorescent staining of neurons on PET membrane showing two axons converging and passing through a single membrane pore. The upper membrane fraction is shown in purple and the lower membrane fraction in green. (**G**) A representative image of two cDNA gel electrophoresis separation quality-control runs; β-actin was used as a positive control and POL-II as a marker for separation. Asterisks indicate samples that passed separation QC and were used for deep sequencing. Sample annotation [XY]: X: C, G, S, T = Control, GFP, SOD, TDP samples, respectively. Y: S = Soma, A = Axon.

An important key to the study of unique subcellular transcriptomes was the ability to isolate RNA from the axon compartment devoid of somatic contaminants. Thus, we have optimized a platform that allows us to mechanically segregate neuronal processes from somas (Fig. 1A). Embryonic spinal cord (SC) cultures were grown for 10 days in a modified Boyden chamber, consisting of a porous membrane that allows passage of axons but restricts the cell body to the upper membrane surface, as previously described^26^. We used dissociated spinal cord motor neuron cultures rather than a spinal cord explant paradigm, which contains many different cell types. This approach provides a cleaner motor neuron preparation, resulting in a more reliable comparison between the somatic and axonal transcriptomes. To validate the separation of the soma from axons, we imaged the top (somatic fraction) and bottom (axon fraction) of the platform in cultures immunostained for neurofilament (NFH) and DAPI (Fig. 1B–E). The imaging data confirms that only neurites grow into the lower compartment, without any contamination of cell bodies. Furthermore, using confocal microscopy we were able to visualize the membrane crossing of axons to the bottom compartment (Fig. 1F). We then assessed the axon-to-dendrite ratio in the bottom compartment by staining for the dendritic marker MAP2 and for Tau as a specific marker for axons (Supplementary Figure 3). These observations confirm that although a small fraction of dendrites do cross into the bottom compartment, it is clearly enriched by axons, hence it will be hereafter considered as the ‘axonal fraction’. After 10 DIV, axons and soma were separately harvested and RNA was extracted. The purity of the separate compartments was validated by RT-PCR followed by agarose gel electrophoresis, using the ubiquitous β-actin and the soma-specific Polymerase II (Fig. 1G). Up to 30 PCR cycles, there was no indication of Polymerase II signal in the axonal compartment; however, some contamination became visible beginning at 35 PCR cycles. This suggests that there are up to 10^4^ molecules of somatic Pol II contamination in the entire axonal fraction, which is considered negligible (0.00067% of the total RNA present within the sample). Pure axonal separation at the RT-PCR level, devoid of any Pol II, was successful in ~50% of the compartmental cultures, and only these ‘pure’ axonal fractions were used for subsequent sequencing and analysis. Total RNA from each fraction (soma/axons was divided into two tubes – one for miRNA NGS and the other for mRNA NGS – allowing direct correlation analyses between mRNA and miRNA profiles.

### Profiling of somatic and axonal RNA

**Figure 2:**
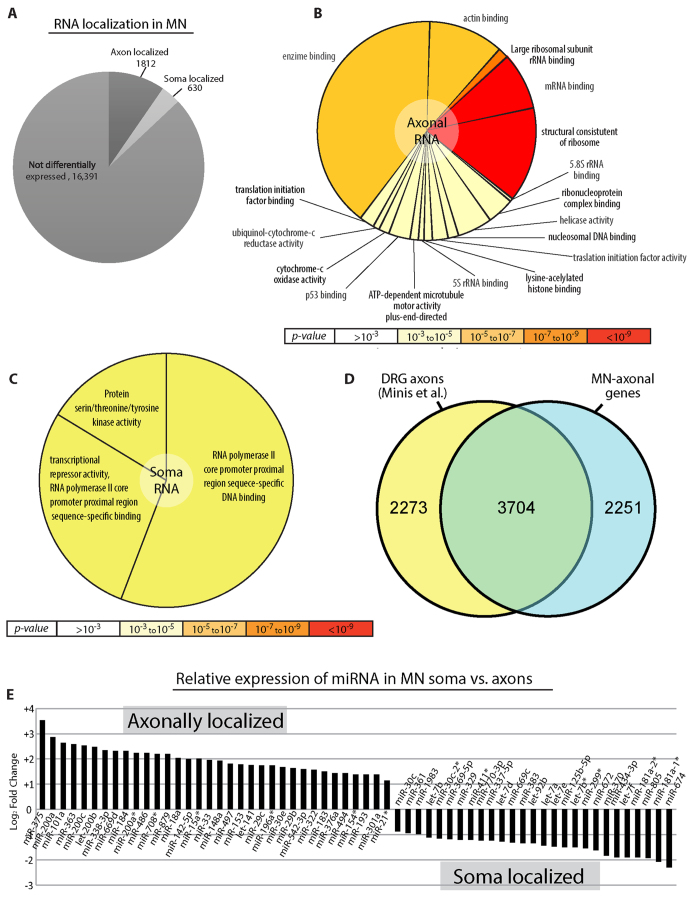
Endogenous axonal RNA. (**A**) Pie diagram of axon-localized RNA species [FC > 2, p-value < 0.05], soma-localized RNA species [FC < 0.5, p-value < 0.05] and RNA not showing differential expression [0.5 < FC < 2 and/or p-Value > 0.05]. (**B**) Axon-localized RNA GO categories. Color is based on significance; the number of genes associated with each category is represented by the size of the pie slice. (**C**) Soma-localized RNA GO categories. Color is based on significance; the number of genes associated with each category is represented by the size of the pie slice. (**D**) Venn diagram comparing all of the genes identified in DRG axons by Minis *et al*. (FPKM > 10) with all of the genes identified from motor neuron axons via deep-seq (normalized expression > 400). (**E**) MicroRNA expression profile within control neurons [axonal: FC > 2, p-value < 0.05, Somatic: FC < 0.5, p-value < 0.05].

We sequenced total RNA from both the somatic and axonal fractions of the MN cultures (n = 3 for each fraction). Sequencing results were mapped using Bowtie2 and Tophat2^27^,^28^. mRNA to miRNA analysis was performed using Partek Genomics Suite software (Version 6.6, Partek Inc., St. Louis, MO, USA). Results were normalized as FPKM (fragments per kilo base pair per million reads mapped) or using the DESeq2 R module^29^. Sequencing of mRNA from the somatic fraction resulted in 18,833 valid genes at various levels of expression (Supplementary Table 1, RawData sheet max/min > 1). False discovery rate (FDR) correction^30^ was applied to all results. This normalization yielded a fold change (FC) value, p-value and *p[FDR]-value* for each gene. Setting the cut off points at absolute value FC > 2, FC < 0.5 and corrected *p-value* < 0.05 produced 2442 differentially expressed genes in axons compared with the soma (Fig. 2A).

As all of our axonally enriched samples were quality controlled using RT-PCR and not western blot analysis, which is less sensitive, it is unlikely that the axonal RNAs that we revealed are a consequence of soma contamination. Notably, upon evaluating most of the mRNAs (16,391), there was no significant difference between the neuronal compartments, suggesting that most mRNAs can also be localized to axons. Still, using these cutoff conditions, 1812 mRNAs were enriched in axons, while 630 mRNAs were soma-enriched (axon-depleted). As the ‘soma compartment’ contains a mixed population of cell bodies, axons and dendrites, the relatively low number of soma-enriched mRNAs can be attributed to the presence of axons in both compartments, that might have statistically blurred and shifted soma localized mRNAs to be associated to the “not differently expressed” group of mRNAs. Nevertheless, the populations of mRNAs that were identified in each compartment represent the most enriched mRNAs in it. To further study the functional enrichment of these lists of genes, we applied the Gene Ontology (GO) analysis tool GOrilla^31^ to specifically characterize the molecular function, biological process and cellular localization using the axon-enriched mRNAs as the target list and the entire 18,833 genes we identified as the background (Fig. 2B and Supplementary Table 2B). This analysis highlighted genes related to functions such as “mRNA binding”, “structural constituent of ribosome”, “actin binding” and “enzyme binding”. These functional groups relate to processes such as “local translation”, the “cytoskeleton” and “metabolic” processes, which have previously been shown to occur in axons (Besse and Ephrussi^21^; Holt and Schuman^3^; Perlson *et al*.^32^). On the other hand, functional analysis of the soma-localized mRNA (Fig. 2C and Supplementary Table 2C) showed enrichment of the RNA polymerase II core promoter, further supporting the quality of the axonal separation procedure. We next compared the axonal-specific mRNA population of our MN cultures to previously described axon-specific mRNAs from dorsal root ganglion (DRG) explants^33^ (Fig. 2D and Supplementary Table 2D). In order to compare a similar number of genes, we used a cutoff of >400 DESeq2-normalized counts. Under these specifications approximately 2/3 of the axonal-enriched mRNAs in the two neuronal populations are shared, while 1/3 are distinct to MNs. Interestingly, GO analysis of these specific MN genes identified gene functions that are related to “receptor activity” and to the process of “projection assembly” and “organization”, “growth”, “chemotaxis”, and “synapse assembly” that are localized to “synapse” and “membrane compartments” (data not shown).

miRNAs can regulate hundreds of genes at the post-transcriptional level by inhibiting mRNA translation or inducing mRNA degradation. Although miRNAs play a crucial role in gene regulation, and increasing evidence points to the localization of miRNAs and RNAi machinery in neuronal compartments such as synapses and axons in order to regulate local synthesis events^34^,^35^, the specific spatial nature of neuronal miRNA populations at the subcellular level has yet to be characterized. Thus, using NGS, we identified 401 miRNAs that exhibit some differential expression between the neuronal compartments. Using FDR correction (Benjamini-Hochberg, corrected *p-value* < 0.05) and cutoff points at absolute value FC > 2, we found that while most of the 401 miRNAs can be found both in the axon and in the soma, 34 miRNAs were specifically enriched in the axons, and 27 were specifically localized in the soma (Fig. 2E). Hence, similar to the mRNA profile, most miRNAs are distributed in both the soma and the axon. Nevertheless, specific miRNAs accumulate in different subcellular locations, suggesting that they might participate in the regulation of restricted metabolic or signaling pathways. Thus, both miRNAs and mRNAs include defined subsets that can be found in distinct neuronal compartments. These transcripts determine the properties of each compartment and contribute to its ability to respond to both external and internal cues.

### Subcellular localization signal motifs

**Figure 3:**
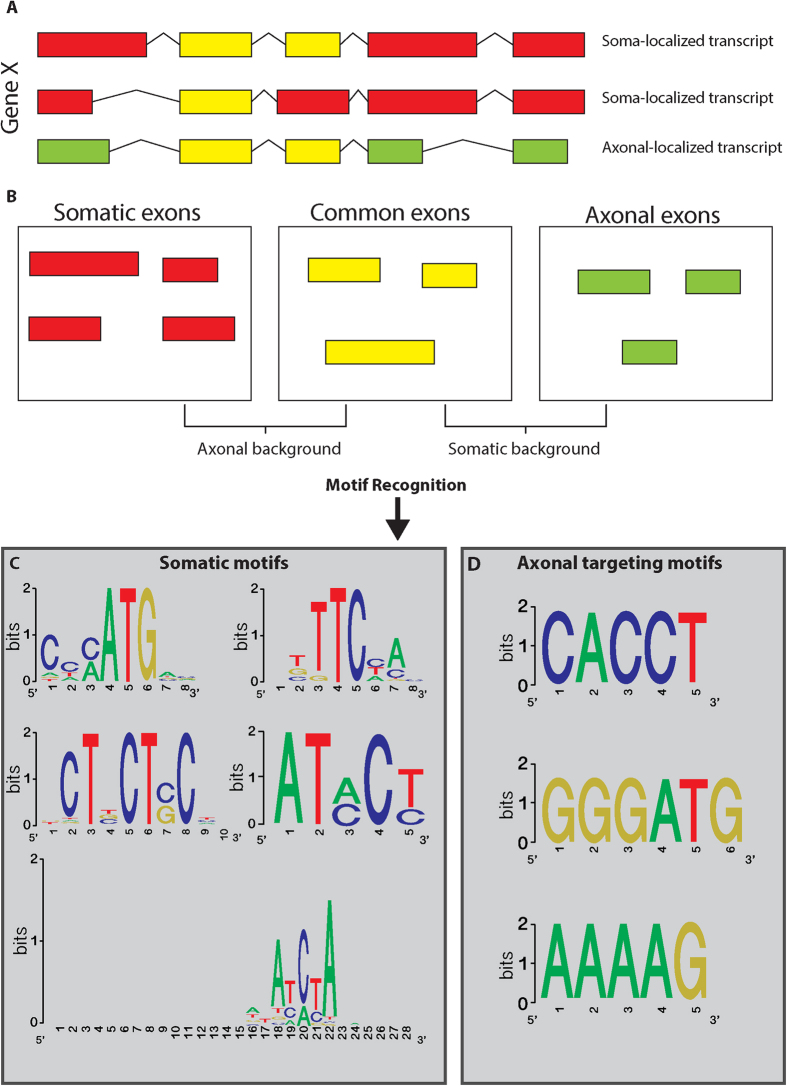
Axonal and somatic localization signals. (**A**,**B**) Schematic representation of the analytical process. Differentially expressed genes with differentially expressed transcripts having at least one transcript expressed in the soma and one in axons were studied. Exons were sorted according to site of expression and used either in the target set or in the background. Target sets were analyzed compared to the background using an online PSSM tool to reveal targeting motifs^68^ (**C**) Soma motifs. (**D**) Axon targeting motifs.

To understand why certain mRNAs are found specifically in the soma while others are enriched in axons, and to study the mechanism of spatial mRNA restriction to distinct subcellular locations, we searched for distinct somatic retention and axonal targeting motifs (Fig. 3). We analyzed 14,658 mRNA transcripts that showed at least a 2-fold difference in soma vs. axon. Among them, 662 mRNAs had more than one transcript isoform. Analyzing motifs in the exons of transcript isoforms localized specifically to the soma or axon, as well as motifs found in isoforms identified in both compartments, we classified unique motifs that may regulate the subcellular localization of specific transcript isoforms (Fig. 3A,B). Five candidate somatic retention motifs (Fig. 3C) and three axonal targeting motifs (Fig. 3D) are suggested as localization motifs. Interestingly, the axonal motifs are much more conserved than the somatic motifs. Further, some of the transcripts contained more than one subcellular motif, while others had multiple copies of the same motif. These motifs are found along the length of the differentially expressed mRNAs and are not exclusively contained within the 3′ UTR, as most of the previously reported targeting motifs have been^36^,^37^,^38^. Furthermore, these motifs are MN-specific and are not similar to motifs previously described in DRGs nor in other publications^33^,^39^,^40^,^41^. Hence, these findings suggest a mechanism in which specific RNA-binding proteins, such as FMRP, TDP43, FUS or Staufen, can travel to the MN axon with distinct, functionally common, RNA populations. This likely involves either binding specifically to these RNA-recognition motifs or recognizing mRNA structural elements, which may be regulated by the number and localization of these motifs along the mRNA. Furthermore, we searched for matches between the RNA-targeting motifs and known RNA Recognition Elements (RRE) of RBP using the “ATtRACT” data-base^42^. We found that while the AAAAG motif is recognized by mouse KHDRBS1 RBP, the CACCT motif is recognized by two other RBPs, YBX2 and YBX3. Our search did not yield any matches for the GGGATG axonal motif.

### RNA profile in the soma of ALS models

**Figure 4:**
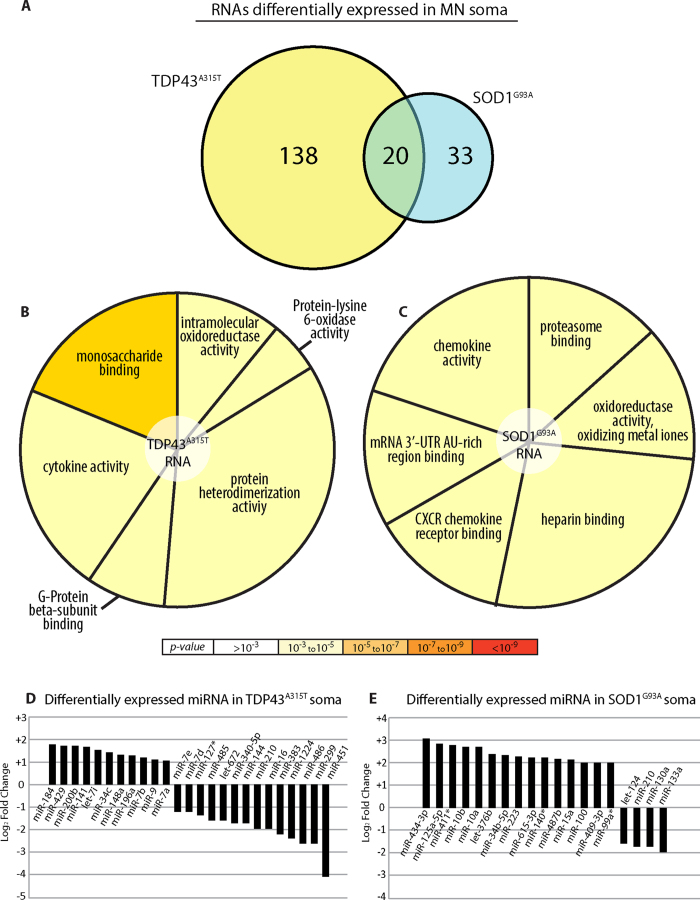
ALS models - somatic RNA. (**A**) Comparing genes showing differential expression in the somas of the SOD1^G93A^ and TDP43^A315T^ ALS models compared to GFP-expressing neurons [above median expression, FC > 2 or FC < 0.5, p-value < 0.05]. (**B**) TDP43^A315T^ differentially expressed genes GO analysis pie chart. Color is based on significance; the number of genes associated with each category is represented by the size of the pie slice. (**C**) SOD1^G93A^ differentially expressed genes GO analysis pie chart. Color is based on significance; the number of genes associated with each category is represented by the size of the pie slice. (**D**) TDP43^A315T^ differentially expressed miRNA compared to GFP-expressing somas (p-value < 0.05). (**E**) SOD1^G93A^ differentially expressed miRNA compared to GFP-expressing somas (p-value < 0.05).

Despite the diverse etiology of ALS, changes in spatiotemporal localization mechanisms are hypothesized to be part of a common toxic mechanism^7^. Thus, the identification of the nature of subcellular motor neuron mRNA and miRNA species in ALS models can be used as the basis for future drug development. Consequently, in order to identify genes that may participate in ALS motor neuron toxicity, we compared mouse primary spinal cord motor neurons expressing GFP::SOD1^G93A^ or GFP::TDP43^A315T^ to GFP controls. These two mutated proteins are thought to cause ALS via different mechanisms and can thus provide comprehensive disease-relevant transcriptome profiles. Interestingly, NGS analysis showed relatively few significant gene alterations in these ALS models compared with the controls (158 in TDP43^A315T^ and 53 in SOD1^G93A^, Fig. 4A and Supplementary Table 4A). This suggests that only specific signaling or metabolic pathways are affected in these ALS models. GO analysis identified altered genes in the TDP43^A315T^ model (Fig. 4B and Supplementary Table 4B) as being associated with a number of categories, including “protein hetero-dimerization activity”, “monosaccharide binding”, “cytokine activity”, “intra-molecular oxidoreductase activity”, “protein-lysine 6-oxidase activity”, and “G-protein beta-subunit binding”. The genes altered in the SOD1^G93A^ soma included genes related to “chemokine activity”, “heparin binding”, “mRNA 3′ UTR binding”, “oxidoreductase activity”, and “proteasome binding” (Fig. 4C and Supplementary Table 4C). We identified 20 genes as being altered in both the SOD1^G93A^ and TDP43^A315T^ models; they may represent a common ALS toxicity pathway. Indeed, GO analysis identified these genes as “binding proteins” that are largely connected to the “regulation of cell death”. Interestingly, though, these gene-products are mainly predicted to localize extracellularly.

In addition, we performed miRNA analyses and identified several miRNAs showing significantly increased or decreased expression in the neurons expressing SOD1^G93A^ (Fig. 4D) or TDP43^A315T^ (Fig. 4E). As miRNAs can regulate the expression of many genes, these alterations may represent key regulators of essential motor neuron signaling and metabolic pathways that are damaged in ALS.

### mRNA profiles in the axons of ALS models

**Figure 5:**
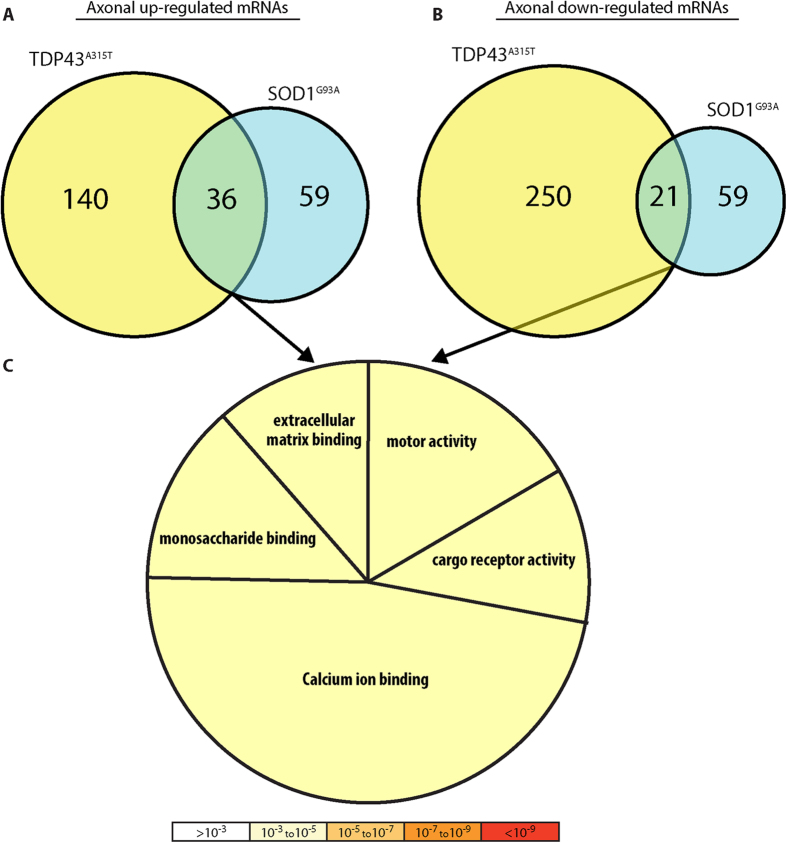
ALS models - axonal mRNA. (**A**) Comparison of genes up regulated (above median expression, FC > 2) in axons of the SOD1^G93A^ and TDP43^A315T^ ALS models compared to GFP-expressing neurons. (**B**) Comparison of genes down-regulated (above median expression, FC < −2) in axons of the SOD1^G93A^ and TDP43^A315T^ ALS models compared to GFP-expressing neurons. (**C**) Pie chart of GO categories associated with differentially expressed genes in **A,B**. Color is based on significance; the number of genes associated with each category is represented by the size of the pie slice.

We next analyzed the compartmental mRNA profiles of the axons of SOD1^G93A^ and TDP43^A315T^ ALS models (Fig. 5). The expression of 176 genes was significantly elevated in the TDP43^A315T^ while 95 were elevated in the SOD1^G93A^ (Fig. 5A and Supplementary Table 5A). The levels of 271 genes in the TDP43^A315T^ and 80 genes in SOD1^G93A^ significantly decreased (Fig. 5B and Supplementary Table 5B). Further, 36 genes were up-regulated and 21 were down-regulated in both the SOD1^G93A^ and TDP43^A315T^ models compared to GFP-infected neurons. GO analysis (Fig. 5C and Supplementary Table 5C) correlated these alterations in ALS axons to “calcium ion binding”, “extracellular matrix binding”, “motor activity”, “cargo receptor activity”, and “monosaccharide binding”. Altogether, we provide for the first time a profile of alterations in the axonal mRNA populations of motor neurons in two ALS models.

### miRNA profiles in the axons of ALS models

**Figure 6:**
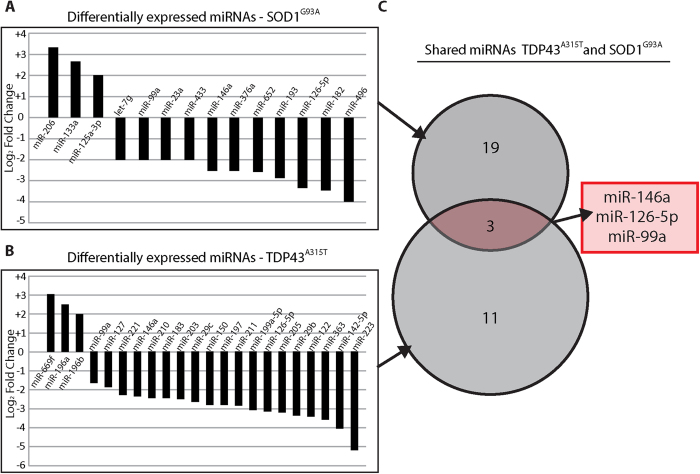
ALS models - axonal microRNA. Differentially expressed microRNA (FC > 2 or FC < −2, p-value < 0.05) logarithmic bar plots in cultures of the SOD1^G93A^ (**A**) and TDP43^A315T^ (**B**) ALS models. (**C**) Venn diagram of microRNAs in A&B. Shared microRNAs are written below the diagram.

Next, we analyzed the comprehensive miRNA profiles of the SOD1^G93A^ and TDP43^A315T^ ALS models, in the axons (Fig. 6). We identified 22 miRNAs as significantly altered in the axons expressing TDP43^A315T^, among which 3 were up-regulated and 19 down-regulated. In the axons expressing SOD1^G93A^, a total of 14 miRNAs were altered compared with the GFP control, among which 3 were up-regulated and 11 down-regulated. miR-146a, miR126-5p and miR-99a were down-regulated in both SOD1^G93A^ and TDP43^A315T^ axons compared to GFP controls (Fig. 6C), indicating that they may serve as key regulators of local synthesis in motor neuron axons, which is altered in ALS.

### Spatial RNA profile in ALS

**Figure 7:**
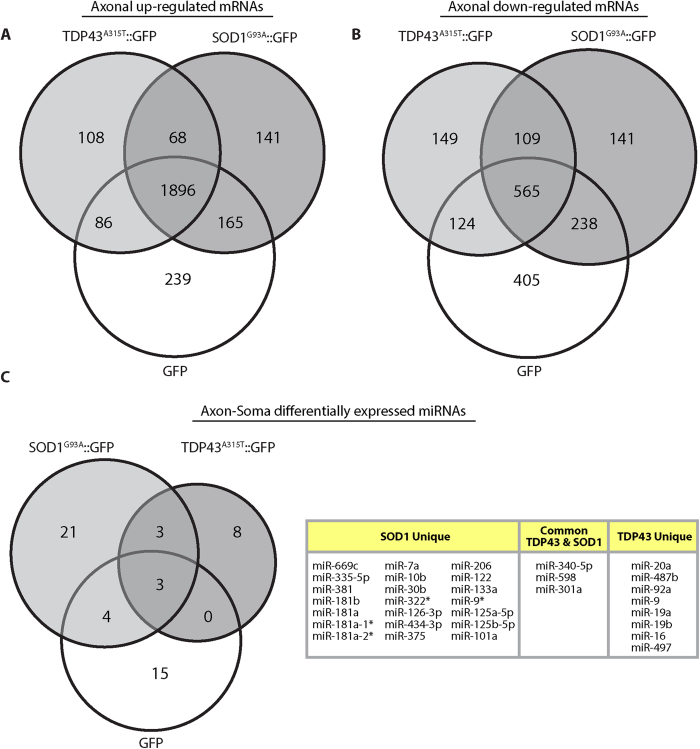
Axon vs. Soma in ALS. (**A**) Venn diagram of genes up-regulated (FC > 2, FDR q-value < 0.05) in axons relative to somas in ALS model cultures compared to axonally up-regulated genes in GFP cultures. (**B**) Venn diagram of genes down-regulated (FC < −2, FDR q-value < 0.05) in axons relative to somas in ALS model cultures compared to genes down-regulated in axons in GFP cultures. (**C**) Venn diagram and table of microRNAs showing axon-soma differential expression (FC > 2 or FC < 0.5, p-value < 0.05) in ALS model cultures compared to differentially expressed microRNAs in GFP cultures.

Given that RNA localization within different neuronal compartments is essential for neuronal health and that there are several mislocalization events of RNA and RNA-binding proteins in ALS^43^, we studied the spatial RNA profiles in ALS models, first comparing the alterations in mRNA localization in the axons vs. the soma between neurons expressing SOD1^G93A^ or TDP43^A315T^ and GFP controls (Fig. 7). We combined the genes showing differential expression between the axons and soma in each model (SOD1^G93A^ and TDP43^A315T^) and our GFP control results. We found that the localization of most mRNAs is not changed (Supplementary Figure 2); however, the localization of 68 mRNAs was shown to be elevated in axons of both ALS models in comparison to control (Fig. 7A and Supplementary Table 7A). Interestingly, GO analysis associated most of these genes to “mitochondria” and as being involved in “metabolic” and “motility processes”. Another 109 transcripts showed decreased localization in the axons of both ALS models (Fig. 7B and Supplementary Table 7B). GO analysis demonstrated the involvement of these genes in processes of “synapse assembly”, “axon extension regulation” and “transcription factor functions”.

Further on, we observed alterations in the localization of several miRNAs (either to soma or axons). 24 miRNAs in the TDP43 model, and 11 miRNAs in the SOD1 model showed an opposite localization compared to their localization in the GFP control. Three miRNAs (miR-340-5p, miR-598 and miR-301a) showed altered localization in the neuronal compartments of both ALS models compared to the GFP control (Fig. 7C). Principal component analysis of transcriptome profiles shows more pronounced mutation-associated disruption of compartmentalization for miRNA compared to mRNA (Supplementary Figure 1).

### mRNA and miRNA correlation analysis

**Figure 8:**
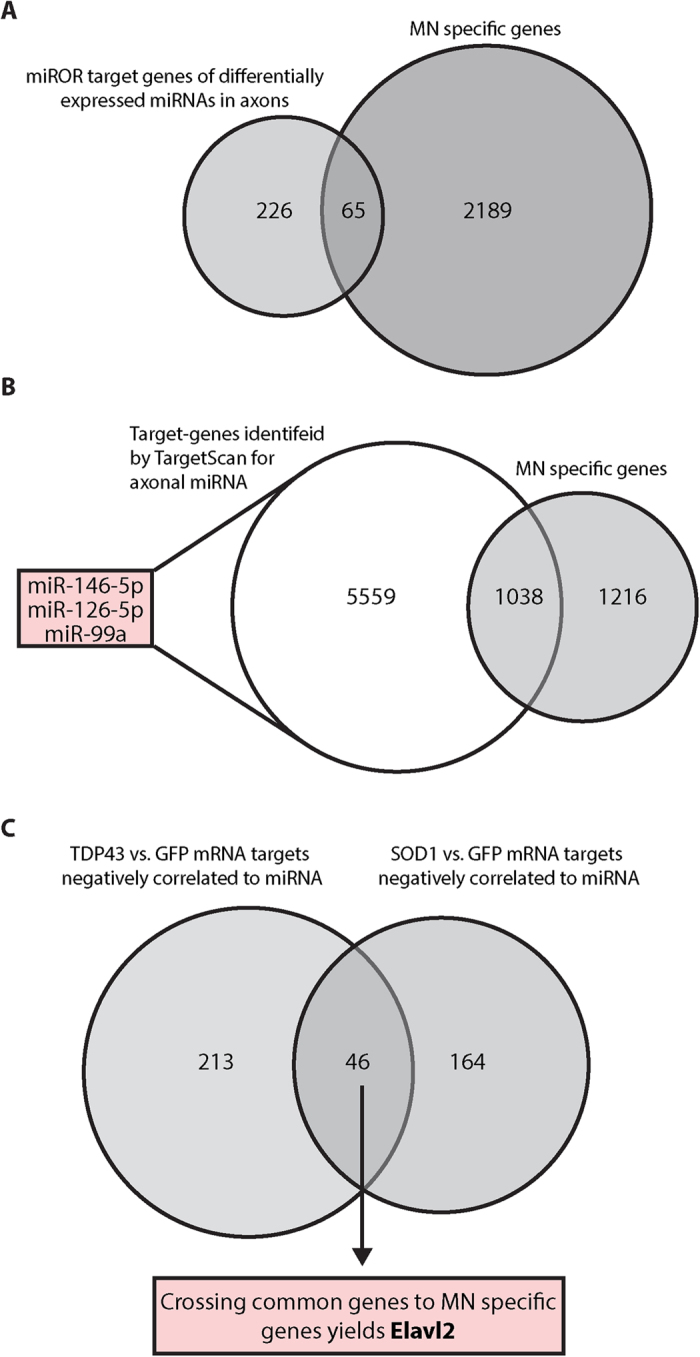
Combined analysis of mRNA and microRNA. (**A**) Venn diagram of the miRror targets of all 33 axonal differentially expressed miRNAs (Fig. 6C) compared with MN-specific genes (Fig. 2D). (**B**) Venn diagram of the TargetScan results for miRNAs showing axonal expression in all three conditions (Fig. 6C) combined with MN-specific genes. (**C**) Venn diagram of negatively correlated genes. Target genes of differentially expressed miRNAs from both models (TDP43^A315T^ and SOD1^G93A^ compared to GFP) were validated as being negatively correlated. Genes showing negative correlations in both lists were reviewed together to reveal 46-shared genes of which Elavl2 is the single MN specific gene.

To further characterize the comprehensive RNA network profile and regulation in the axons, we used the miRror analysis tool^44^ to predict targets of the 33 miRNAs that were altered in the axons of both SOD1^G93A^ and TDP43^A315T^ ALS models (Fig. 6A,B), identifying 291 genes that are targeted by all of the miRNAs above. As ALS is a MN disease, we wanted to find MN-specific alterations. Evaluating this list of predicted targets in combination with the specific mRNAs that were found in MN axons but not in DRGs (Fig. 2D), we identified 65 genes potentially representing the important signaling and metabolic regulatory network(s) of motor neurons (Fig. 8A and Supplementary Table 8A). GO analysis of these genes identified genes connected to “apoptotic and necroptotic signaling pathways”, “ion channels”, and “CNS development”.

We additionally used TargetScan^45^,^46^ to predict targets for each of the common miRNAs found (miR-146-5p; miR-126-5p; miR-99a, Supplementary Data Tables). Using this tool, we identified approximately half of the mRNAs enriched in motor neuron axons (1038 genes) as predicted targets of at least one of these miRNAs (Fig. 8B and Supplementary Table 8B).

Next, the predicted targets of axonally altered miRNAs of the two ALS models (122 miRNAs in TDP43 and 83 miRs in SOD1) were correlated to mRNAs showing differential axonal expression in the ALS models. In axons of the TDP43 model, we identified 259 mRNAs as negatively regulated compared to the differentially expressed miRs. Similarly, 210 mRNAs were identified in the SOD1 model. Among them, 46 of the mRNAs were common to both models (Fig. 8C and Supplementary 8C Tables). Comparing these common genes to MN-specific genes (Fig. 2D) yielded only the RNA-binding protein Elavl2 (Fig. 8C). Thus, Elavl2 is a differentially expressed MN-specific axonal mRNA whose expression can be regulated by axonal miRNAs that are altered in both ALS models.

### Identification of Elavl2 mRNA and protein in MN

**Figure 9:**
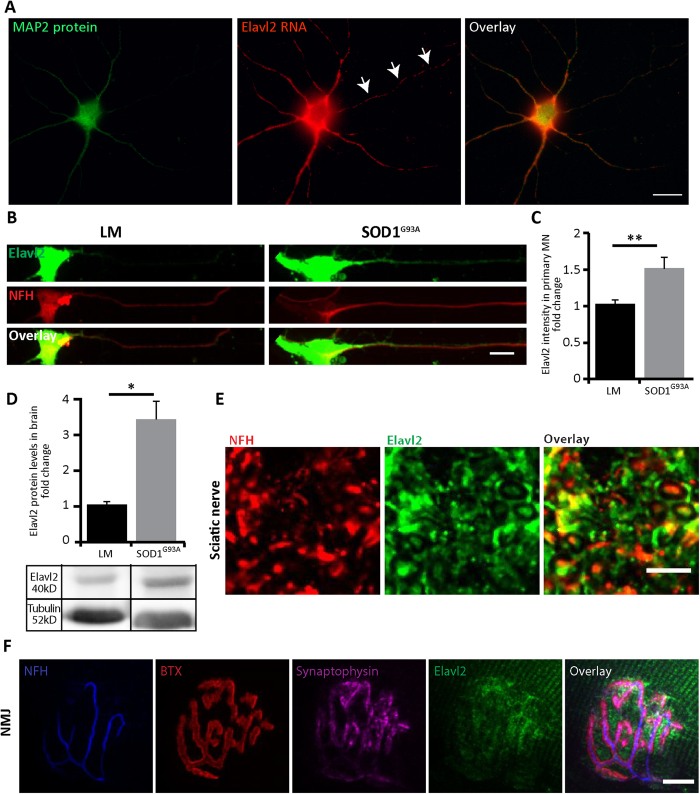
*In-Vitro* and *In-Vivo* Characterization of Elavl2 Expression in MN. (**A**) *In situ* hybridization for Elavl2 mRNA in primary MN cultures from wild-type mice shows abundant mRNA in the soma and MAP2-positive dendrites. In addition, distinct puncta are present along the MAP2-negative axon shaft (arrows). Scale bar: 20 μm. (**B**) Immunofluorescence staining for Elavl2 in primary MN cultures from SOD1^G93A^ and LM mouse embryos show increase in the axonal expression of Elavl2 in SOD1^G93A^ MN. Green color indicates Elavl2, red color indicates NFH Scale bar: 10 μm. (**C**) Quantification of Elavl2 immunofluorescence intensity in axons only reveals a significant ~1.5 fold increase in the protein levels of Elavl2 in axons of SOD1^G93A^ over LM controls. **p < 0.01 (student’s t-test; n = 61). (**D**) Western blot analysis of Elavl2 levels in brains of postnatal day 120 (P120) SOD1^G93A^ and LM mice shows a significant increase in Elavl2 levels. Tubulin was used as loading control. *p < 0.05 (Student’s t-test, n = 3). The blot was cropped, and its full length is presented in Supplementary information. (**E**) Whole mount immunofluorescence staining of gastrocnemius muscle from P60 SOD1^G93A^ for Elavl2 protein reveals enrichment its levels at the neuromuscular junction region, as shown by its co-localization with pre- and post-synaptic markers. Blue color indicates NFH in presynaptic MN. Red color indicates BTX staining for postsynaptic AchR aggregation. Green color indicates Elavl2. Magenta color indicates Synaptophysin in the presynaptic MN. Scale bar: 10 μm. (**F**) Immunofluorescence staining for Elavl2 in sciatic nerve cross-sections shows the presence of Elavl2 protein in axons. Red color indicates NFH, Green color indicates Elavl2. Scale 10 μm.

As our predictions suggest that Elavl2 mRNA is localized in axons of MN, we sought to characterize its protein and mRNA localization. To this end we performed Fluorescent *In-Situ* Hybridization (FISH) in cultured primary MN in order to detect Elavl2 mRNA in axons (Fig. 9A). Our observations confirm that Elavl2 mRNA is localized into MN axons, as it is present also in neuronal processes missing the MAP2 protein. Next, we wished to determine the nature of Elavl2 protein in healthy and SOD1^G93A^ diseased MN axons. 7-DIV primary MN cultures from E12.5 SOD1^G93A^ and littermate (LM) mouse embryos were fixed and immunostained for Elavl2 protein (Fig. 9B). Quantification of the fluorescence intensity exclusively in axons revealed a significant 1.5 fold increase in Elavl2 in axons from SOD1^G93A^ background over their littermate controls (Fig. 9C; LM 1.00 ± 0.06; SOD1^G93A^ 1.49 ± 0.15; data is shown as mean fold change over LM ± SEM). Following the identification of Elavl2 alterations in MN we questioned whether its levels are also affected *in-vivo* in adult mice. Brains from adult P120 SOD1^G93A^ and LM mice were excised, homogenized and the levels of Elavl2 were analyzed by western blot. Concomitantly to our observations *in-vitro*, western blot quantification shows a similar elevation in Elavl2 protein in brains of SOD1^G93A^ compared to their LM (Fig. 9D; LM 1.00 ± 0.01; SOD^G93A^ 3.25 ± 0.08; results are shown as mean fold change over LM ± SEM. Next, in order to verify that Elavl2 is also localized in MN axons *in-vivo,* we isolated the sciatic nerves of adult mice and performed cryosectioning and immunostaining for Elavl2 and NFH (Fig. 9E). Indeed, we were able to observe co-localization of Elavl2 and NFH in sciatic nerve cross-sections, indicating Elavl2 is found in distal axons. Elval2 was previously suggested to have an important role in presynaptic differentiation in granule neurons^47^. Considering our findings that show Elavl2 is localized in MN axons, we wished to determine whether it is present also at the pre-synapses of MN, the Neuromuscular Junction (NMJ). Gastrocnemius muscles were excised from adult mice and immunostained for Elavl2 and synaptic markers of the NMJ (Fig. 9F). Our results reveal that Elavl2 protein is enriched at the NMJ and is co-localized with pre-synaptic Synaptophysin and NFH, thus suggesting a novel role for this protein in MN. Altogether, our data shows that Elavl2 mRNA is localized to MN axons and its protein might have a spatial-related role in regulation on neuromuscular junction maintenance. Together with these findings, the alterations we observed in Elavl2 protein and mRNA levels in SOD1^G93A^ mice propose it may have a contribution to the NMJ disruption that occurs in ALS in a yet to be characterized mechanism.

## Discussion

Local protein synthesis is essential for the development, survival, function and plasticity of neurons as they respond to external cues and various stimulations^3^,^39^,^48^,^49^,^50^. Indeed, alterations in local protein synthesis can be toxic to the neurons and has been associated with motor neuron diseases^6^,^7^,^51^. In the current study, we examined the MN subcellular transcriptome of primary motor neuron of two ALS models, allowing us to determine whether the mRNA and miRNA milieus change and whether they have alterations in common. Many of the mRNAs and miRNA species in the MN axon are associated with diverse functions and metabolic processes, suggesting that the axonal compartment can maintain its own specific homeostasis in altering microenvironments largely by local protein synthesis that may be regulated also by miRNAs. Thus, the ability to regulate and control local protein synthesis might be associated with the disease mechanism and/or the ability to respond to spatiotemporal stress (axon/soma), leading to higher vulnerability in ALS.

### The axonal transcriptome

Several previous studies have analyzed neuronal subcellular RNA localization^33^,^52^,^53^,^54^,^55^,^56^,^57^,^58^. Here, for the first time, we comprehensively characterized the RNAs (both mRNAs and microRNAs) expressed in motor neuron axons, and we further investigated the nature of axonal RNAs that are altered in two ALS models that are believed to act via different mechanisms. These unique RNA profile signatures may play a role in axon and NMJ maintenance, which are disrupted in ALS. Our analyses identified mRNAs of which localization has shifted in SOD1^G93A^ and in TDP43^A315T^ motor neurons coordinately compared to the GFP control, raising the possibility for shared features in the disease mechanism of both models. Interestingly, the genes that undergo enrichment in axons of both SOD1^G93A^ and TDP43^A315T^ associate with the mitochondria, of which function was previously described to become impaired in several ALS models^59^. Furthermore, mRNA localization to axons and its local translation were shown to be critical for the proper function of mitochondria in axons^50^. Therefore, it will be of high interest to further study the nature of these mRNAs and their contribution to motor neuron death in ALS. Although we have gained some knowledge over the last few years about the role of local RNA translation in synapse formation (for review see Bramham and Wells^60^) or in response to axonal injury^5^,^61^, information about the roles played by axonal mRNAs and miRNAs in intact motor neurons under basal or pathological conditions such as ALS is scarce.

Setting the cutoff to obtain numbers of mRNA transcripts in MN axons (5,955) similar to the previous report for DRG axons (5,977^33^), we demonstrate that approximately 60% of the axonal transcripts are common to both DRG and MN axons, suggesting that these are broadly expressed in sensory and motor neurons alike. These abundant and commonly expressed axonal mRNAs encode for proteins that participate in metabolic and signaling processes and also include RNA-binding, transport, translation and protein modification activity. Still, however, approximately one third of the expressed RNAs are unique, suggesting cell subtype-specificity. This may indicate the existence of cell-autonomous properties that clarify the physiological differences between sensory and motor neurons. Among the unique MN axonal mRNAs, the highest enrichment was observed for mRNAs associated with “receptor activity”, “axon growth”, “chemotaxis”, and “synapse assembly”. Furthermore, comparing the MN and DRGs RNA population suggests that defined mRNA repertoires might determine distinctive properties, such as vulnerability to stress. Lastly, in agreement with recent models in which most of the neuronal proteome can be controlled by local translation^3^, our work identified miRNAs that can regulate these local translation events.

### Axonal and somatic mRNA motifs

We attempted to search for consensus sequences in the divergent somatic and axonal RNA populations as means of identifying characteristics pointing to common function(s). We identified putative axonal and somatic mRNA motifs, supporting the idea that specific mRNA splice variants target the neuronal subcellular compartments. GO enrichment analysis for the genes containing axonal targeting motifs identified the following gene categories: RNA metabolic processes, transcription and gene expression, splicing, DNA damage and repair, methylation, neuron projection, regulation of neuron death, neuron differentiation and regulation of neurogenesis. Importantly, the set of short sequences that we found differs from those found in DRGs as well as several other previous works, possibly indicating cell-type-specific regulation. The biological roles and mechanisms of these MN subcellular motifs have not yet been determined. It will be interesting to determine whether specific signaling cues, RNA-binding proteins, or RNA secondary structure contribute to the sorting of mRNAs that harbor common sequence motifs. This may be a means of segregating these RNAs into defined RNA groups, either to direct them into specific cargo granules for transport along the axon, to seed match binding-sites for miRNA regulation, or to coordinate their function at precise subcellular region.

### Axonal transcriptome changes in ALS models

This work provides a comprehensive resource for metabolic and signaling components that are altered in ALS models. Notably, most genes that were altered in the SOD1 model were not altered in the TDP43 model, and vice versa. This is also consistent with the ALS pathology, which is distinguishable between the fALS SOD1 phenotype and the fALS TDP43 phenotype. Only a few genes are altered in both ALS models. Thus, different pathways are likely activated in each model, culminating in similar neuronal toxicity and resulting in an ALS phenotype. Still, it will be important to study the common pathway and determine whether manipulating it can produce beneficial effects in both models.

As ALS is a multifactorial disease that includes alterations in several metabolic and signaling pathways, master regulators such as miRNAs can serve as drug targets. In our screen, we found several miRNAs that can be used in the future for functional assays. Importantly, we identified the RNA-binding protein Elavl2 as a possible key player in axonal health. Elavl2 expression was almost unchanged within the soma, but it was elevated in the axons of both the SOD1 and TDP43 models. Furthermore, we were able to localize Elavl2 in sciatic nerve axons and observed its enrichment at the NMJ. It has been previously reported that Elavl1, 2 and 3 contain RNA Recognition Motifs (RRMs) and prion-like domains and that these proteins aggregate and can be toxic in yeast, mimicking their behavior in disease and similar to what is observed with FUS and TDP43^62^. Interestingly, the reverse sequence of the AAAAG axonal motif (GAAA) that we have identified, is recognized by Elavl2, and suggests Elavl2 might also target RNAs containing the AAAAG motif into axons, depending on the specific recognition mechanism between the protein and the RNA motif. Moreover, Elavl2 was found as a rare variant in sporadic ALS patients (in 1000 genomes or dbSNPv137 databases). Thus, in the future, it will be important to determine the specific functions and roles of Elavl2 in MN and more specifically in ALS toxicity.

In summary, we provide here for the first time a comprehensive subcellular evaluation of the motor neuron transcriptome, both in healthy and diseased neurons. As the evidence accumulates for the importance of subcellular localization of RNA, RNA-binding proteins and, particularly, miRNAs in health and disease, the transcriptome datasets presented here will be of great value for investigating the biology of motor neurons and for investigating new treatment approaches for ALS and other neurological disorders.

## Experimental Procedures

All methods were carried out in accordance with Tel-Aviv University guidelines and regulations. All animal experimentations were approved by Tel-Aviv University Animal Ethics Committee.

### “Modified Boyden chamber” membrane platform

Membrane inserts with 1 μm pores (Falcon^®^ Permeable Support for 6 well plates features transparent PET membrane with 1.0 μm pores, Corning) were placed in a 6-well plate (Corning). The membranes were coated first by filling the membrane insert (2 ml) and corresponding well (3 ml) with poly-DL-ornithine (1.5 μg/ml, Sigma-Aldrich) overnight at 37 °C then with Laminin (3 μg/ml, Sigma-Aldrich) for two hours at 37 °C prior to cell plating.

### Dissociated spinal cord culture

Neuronal cell culture was performed according to “Rapid purification of embryonic rat motor neurons: an *in vitro* model for studying MND/ALS pathogenesis”^63^. Briefly, spinal cords were collected from ICR mice (Harlan) at E12.5. Spinal cords were then dissociated using trypsin and repeated trituration. Cells were collected through centrifugation followed by Optiprep (Sigma-Aldrich) gradient centrifugation to achieve a motor neuron-enriched cell culture. Neurons were then either plated on the coated membrane inserts (500,000 cells per insert) with complete Neurobasal medium or subjected to the lentiviral infection protocol^64^. All neuronal cultures were grown for 10 DIV.

### Lentivirus infections

Viruses containing mutations known to be associated with ALS were generated by transfecting HEK293T cells using a calcium-phosphate transfection protocol with second generation Gag-Pol, VSVG and the target vector. Growth medium was collected 48 hours after transfection and concentrated using a PEG virus concentration kit (Abcam). Neurons were then infected with 1 MOI of virus in 0.5 ml of medium and rotated at 37 °C for two hours before plating on coated membrane inserts. Uninfected control cultures were similarly rotated and incubated at 37 °C for two hours.

### Axonal harvesting

After 10 DIV, each culture was washed by gently replacing the growth medium with PBS warmed to 37 °C. Each culture well was then evaluated visually for GFP expression levels. Wells showing infection levels <70% were discarded. The remaining wells were subjected to two further 37 °C PBS washes. After the third wash, PBS was aspirated away, and the bottom part of the insert (axonal domain) was scraped using a sterile swab tip (TX761MD, Tex-wipe). The scraping tip of the swab was then cut and placed into Qiazol reagent (Qiagen) for 1–2 min, before the Qiazol reagent was transferred into a new collection tube. The inner part of the membrane (somatic domain) was repeatedly washed with 0.7 ml of Qiazol and placed into a collection tube.

### Spin-column total RNA extraction

RNA from both the somatic and axonal domains was extracted according to the miRNeasy micro kit protocol (Qiagen) using the specific protocol for small amounts of RNA for the axonal fraction and applying on-column DNase for all samples.

### Reverse transcription-PCR

Total RNA was diluted to 0.3 ng/μl and reverse transcribed into cDNA using the SuperScript first-strand synthesis system (Invitrogen). Then, 3 ng of cDNA was amplified in a C1000 duel thermal cycler (Bio-Rad) according to the KAPA 2 G Fast PCR Kit protocol (KAPA Biosystems) and using the appropriate primers and temperatures for β-Actin (FWD-GTATGGAATCCTGTGGCATC; REV-AAGCACTTGCGGTGCACGAT; 55 °C) and Polymerase B (FWD-CCAAGGACAGGAAGTGAATGAC; REV-AAGCACAGAGAAGAGGCAATC 52 °C) (IDT).

### mRNA library preparation

Library preparation for all mRNA samples was performed by the University of Minnesota Genomic Center (UMGC) following the TruSeq RNA v2 library preparation protocol (Illumina).

### miRNA library preparation

For small RNA cDNA library preparation 3′ pre-adenylated barcoded adapters (1.25 μM) were ligated to 0.5–10 ng of total RNA with 0·05 μg/μL mutated-truncated Rnl2 ligase (New England Biolabs, Whitby, ON, Canada), in the presence of ten 22-nt synthetic non-human spike-in controls for absolute miRNA quantification. Then, 24 3′-ligated samples were pooled to generate a single multiplexed library, separated by polyacrylamide-urea gel electrophoresis, excised and eluted overnight at 4 °C. Then, a 5′ adapter, 5′-GTTCAGAGTTCTACAGTCCGACGATC-3′, was ligated (T4 Rnl1 ligase; New England Biolabs) to each fragment, and products were separated by gel electrophoresis, excised, eluted overnight at 4 °C, and precipitated with 3 volumes of 100% ethanol and 3 μl glycogen. Reverse transcription was performed with an RT primer 5′-CCTTGGCACCCGAGAATTCCACAGAGGACAGCATACGA-3′ and Superscript III RT (Life Technologies, Carlsbad, CA), and the cDNA library was amplified with ReadyMix^™^ Taq PCR Reaction Mix. The 3′ primer 5′-CCTTGGCACCCGAGAATTCCACAGAGGACAGCATACGA-3′ was used for cDNA amplification, and 5′-AATGATACGGCGACCACCGACAGGTTCAGAGTTCTACAGTCCGA-3′ was used as a 5′ primer. The cDNA library underwent 60 cycles of single-read sequencing (HiSeq 2500, Illumina, San Diego, CA).

### mRNA sequencing

All mRNA samples were sequenced at the UMGC. Due to very low levels of RNA, 3 axonal samples per treatment were combined into one pooled sample before sequencing. A total of 16 samples were divided into two groups. Eight samples were sequenced using a HiSeq 2000 (Illumina) 100 bp paired-end single lane run. The remaining eight samples were split into two HiSeq 2000 (Illumina) 50 bp paired-end sequencing lanes.

### Post-sequencing processing and alignment for mRNA

Sequencing data were evaluated using FastQC software (Barbraham Institute). Sequencing reads were trimmed using fastq-mcf (ea-utils, Aronesty^65^) to remove low-quality base reads (Phred score < 30) in addition to removing sequencing adapter reads. All remaining reads were verified to be longer than 15 nt. Trimmed reads were than aligned to UCSC’s mm10 genome and transcriptome using Tophat 2^27^ and Bowtie 2^28^.

### Post-sequencing processing and alignment for miRNA

The obtained small RNA sequence files were trimmed and split into the separate samples according to the barcode sequences^66^. Extracted reads were assigned annotations by alignment to the genome and small-RNA databases. For miRNA annotation, we used current in-house definitions^67^.

### miRNA target prediction

The miRror suite^44^ and TargetScan 7.0^45^,^46^ were used for predicting miRNA targets.

### Immunofluorescent staining of MN

MN cultures were washed with warm 1X PBS and immediately fixed in 4% PFA for 15 minutes. Samples were permeabilized in 0.5% triton for 10 minutes and blocked in blocking solution (1 mg/mL BSA; 10% Goat serum; 0.1% triton in PBS) for 1 hour at room temperature. Samples were incubated with primary antibodies in blocking solution for 12 hours at 4 °C. Antibodies were used in the following concentrations: Elavl2 1:50 (Rabbit; Proteintech, 14008-1-AP), NFH 1:500 (Chicken; Abcam, ab72996) 1:1,000 MAP2 (Rabbit; Millipore, AB5622), 1:100 Tau (Mouse; Abcam, ab80569). Samples were then incubated for 2 hours with fluorescent secondary antibodies: DyLight 405 anti-chicken 1:200; Alexafluor 488 anti-rabbit 1:500; AlexaFluor 647 anti-mouse 1:500; AlexaFluor 594 anti-rabbit 1:500. Samples were mounted using ProLong^®^ gold antifade reagent.

### Neuromuscular Junction Staining

Gactrocnemius muscle was excised, dissected into smaller sections, and fixed in 4% paraformaldehyde, and then incubated with 1 μg/mL Rhodamine Red-Conjugated Bungarotoxin (Sigma-Aldrich). Tissues were then treated with methanol at −20 °C for 5 min, and afterwards blocked in blocking solution (1 mg/mL BSA, 0.1% triton in PBS) for 1 hour. Tissues were then rocked with appropriate primary antibodies diluted in blocking solution at room temperature overnight. Antibodies were used at the following concentrations: NFH 1:500 (Chicken; Abcam, ab72996); Elavl2 1:50 (Rabbit; Proteintech 14008-1-AP); Synaptophysin 1:500 (Mouse; millipore, mab5258) After washing, secondary antibodies (DyLight 405 anti-chicken 1:500; AlexaFluor 488 anti-Rabbit 1:500; AlexaFluor 647 anti-mouse 1:500) were added for 4 hours at room temperature. Muscle fibers were spread into monolayers under a stereomicroscope and affixed to slides using VectaShield (Vector Laboratories).

### Sciatic nerve immunostaining

Sciatic nerves were excised of adult mice and incubated in 4% PFA for 12 hours at 4 °C, followed by 1X PBS washes and 12 hour incubation in 20% Sucrose. Sciatic nerves were embedded in OCT and frozen for cryosectioning into 10 μm slices. Samples were then washed and permeabilized in 0.1% triton, 1 mg/mL BSA, 10% goat serum in PBS. Primary antibodies were incubated in blocking solution (1 mg/mL BSA, 10% goat serum) for 12 hours. Antibodies were used at the following concentrations: NFH 1:500 (Chicken; ab72996); Elavl2 1:50 (Rabbit; 14008-1-AP). After primary antibodies were washed, samples were incubated with secondary antibodies for 2 hours at room temperature (DyLight 405 anti-chicken 1:200; AlexaFluor 488 anti-Rabbit 1:500). Samples were mounted using ProLong^®^ gold antifade reagent.

### Fluorescence microscopy and image analysis

All confocal images were captured using a Nikon Ti microscope equipped with a Yokogawa CSU X-1 spinning disc and an Andor iXon897 EMCCD camera controlled by Andor IQ2 software. Images were analyzed using ImageJ software.

### Western Blot

Brain tissue was excised and homogenized in lysis buffer containing PBS, 1% Triton X-100 (Sigma), and 1% protease inhibitors (Roche), followed by centrifugation and collection of the supernatant. Protein concentration was determined using the Bio-Rad Protein Assay. Protein samples were denatured by boiling in SDS sample buffer and then electrophoresed in 10% polyacrylamide gels (SDS-PAGE). Proteins were transferred to a nitrocellulose membrane and then immunoblotted with appropriate primary antibodies: Elavl2 1:1,000 (Rabbit; Proteintech, 14008-1-AP); Tubulin-α 1:10,000 (mouse; Abcam, ab7291) diluted in 5% (w/v) Skim-milk (BD Difco) in TBS-T, followed by species-specific HRP-conjugated secondary antibodies (Jackson Laboratories) and visualized using a myECL imager (Thermo), according to the manufacturer’s instructions. Quantification was performed using ImageJ software.

### *In-Situ* hybridization

Detection of Elavl2 mRNA was performed using commercially available RNAscope probes following the manufacturers protocol (Advanced Cell Diagnostics). The standard protocol was modified to allow for combined FISH and immunofluorescent staining for motor neuron markers. Briefly, E12.5 motor neurons were plated on coated coverslips and allowed to grow for 7 days. Neurons were fixed with 4% paraformaldehyde for 20 minutes at room temperature and boiled for 10 minutes in antigen retrieval solution (10 mM Sodium Citrate, 0.05% Tween in 1X PBS) to allow for eventual IF staining. They were then hybridized to a probe recognizing Elavl2 (Mm-Elavl2 targeting 1909-3474 of NM_001347149.1) according to the RNAscope Multiplex Fluorescent Assay for Cultured Adherent Cells protocol (ACD, 320850). All incubation steps were performed at 40 °C within the HybEZ hybridization oven. MAP2 protein was detected by immunofluorescence using mouse anti-MAP2 (1:100, Millipore MAB3418) followed by AlexaFluor goat anti-mouse 488 (1:1,000, Invitrogen). All images were collected using a Zeiss 510 scanning confocal microscope.

## Additional Information

**How to cite this article:** Rotem, N. *et al*. ALS Along the Axons – Expression of Coding and Noncoding RNA Differs in Axons of ALS models. *Sci. Rep.* **7**, 44500; doi: 10.1038/srep44500 (2017).

**Publisher's note:** Springer Nature remains neutral with regard to jurisdictional claims in published maps and institutional affiliations.

## Supplementary Material

Supplementary Figures

Supplementary Table
