# Supplementary material for: ALS Along the Axons – Expression of Coding and Noncoding RNA Differs in Axons of ALS models
Source: Sci Rep. 2017 Mar 16;7:44500. doi: 10.1038/srep44500 (PMC5353576; doi:10.1038/srep44500)

ALS Along the Axons – Expression of Coding and Noncoding RNA Differs in Axons of ALS models

Nimrod Rotem, Iddo Magen, Ariel Ionescu, Noga Gershoni-Emek, Topaz Altman, Tal Gradus, Metsada Pasmanik-Chor, Dianna E. Willis, Iddo Z. Ben-Dov, Eran Hornstein and Eran Perlson

Supplementary figure legend:

Supplementary figure 1 - miRNA and mRNA PCA plots
Principal component analysis reveals different patterns of mutant-induced spatial transcriptome alterations. Compartmental transcription profiling was performed using RNA sequencing. Axon specimens were pooled for mRNA profiling, while individual (non-pooled) samples were sequenced for Soma mRNA and Axon and Soma miRNA. Principal component analyses were performed following TMM normalization of count data (Robinson et al. 2010) followed by voom transformation (Ritchie et al. 2015). The plots display principal component (PC) 2 vs. PC1 for miRNA (**top panel**) and mRNA (**lower panel**). Mutant genotypes seem to disrupt the Axon-Soma compartmentalization of miRNA profiles, but not of mRNA profiles.

Supplementary figure 2: Axon vs. Soma MA Plots
MA plots showing axons vs. soma mRNA expression ratios (log2 fold change) in Control (upper left), GFP (upper right), TDP (lower left) and SOD cells (lower left). mRNA raw counts (excluding mRNA not detected in at least 50% of samples) were subjected to a DESeq2 model with genotype, cell fraction and the interaction term as predictor variables. The MA plots represent results extracted from this model. The pattern of mRNA differential expression (fractionation) is grossly similar across the genotypes.

Supplementary figure 3: Axons vs. dendrites proportions in the modified Boyden chamber compartments. Immunostaining for MAP2 (Red; marks dendrites) and Tau (Green; marks axons) and DAPI (Blue) show homogeneous distributions of axons and dendrites in the upper side of the modified Boyden chamber where cell bodies are found (upper panel) compared to axonal enriched distribution on the bottom side, which staines negatively also for cell bodies (lower panel). Scale bar: 30µm.

Supplementary figure 4: Elavl2 protein levels are increased in P120 brains of SOD1^{G93A} mice compared to their LM. Full length blots of figure 9D showing increase in 40kD Elavl2 protein levels in brains of SOD1^{G93A} mice compared to their LM. Upper blot shows Elavl2 bands. Lower blow shows 55kD Tubulin loading control.

Ritchie, M.E. et al., 2015. limma powers differential expression analyses for RNA-sequencing and microarray studies. *Nucleic acids research*, 43(7), p.e47.
Robinson, M.D., McCarthy, D.J. & Smyth, G.K., 2010. edgeR: a Bioconductor package for differential expression analysis of digital gene expression data. *Bioinformatics (Oxford, England)*, 26(1), pp.139–40.

Supplementary Figure 1

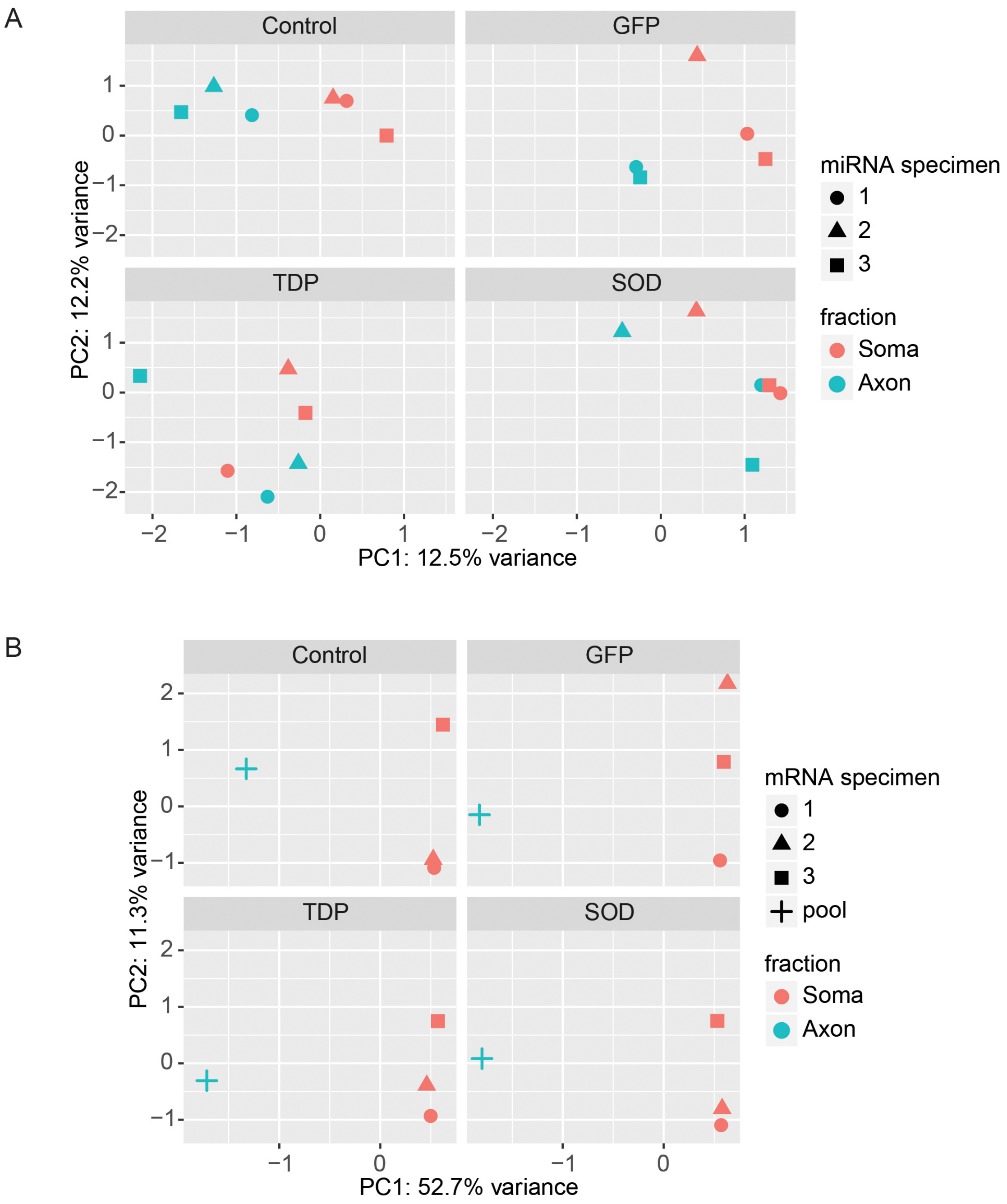

Supplementary Figure 2

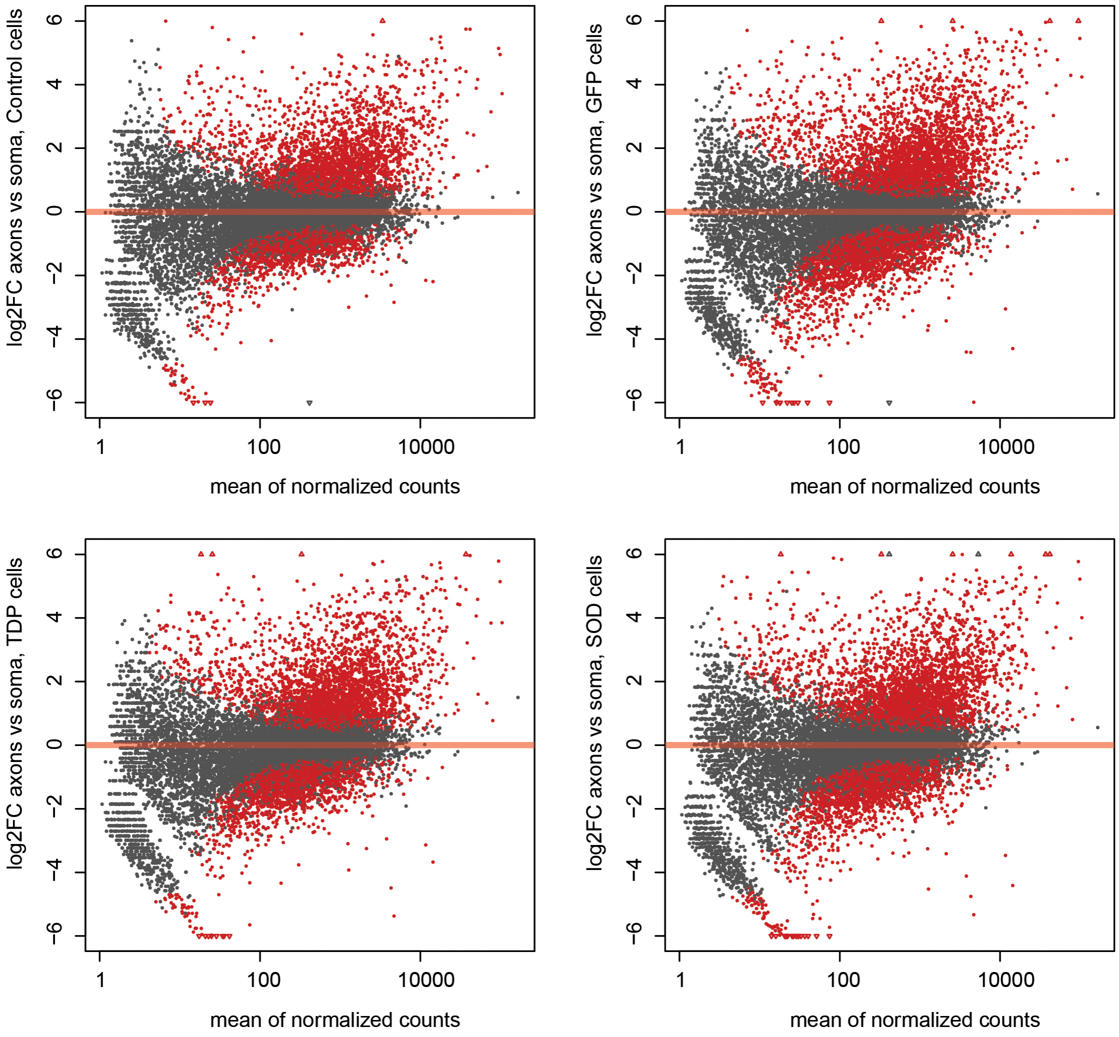

Supplementary Figure 3

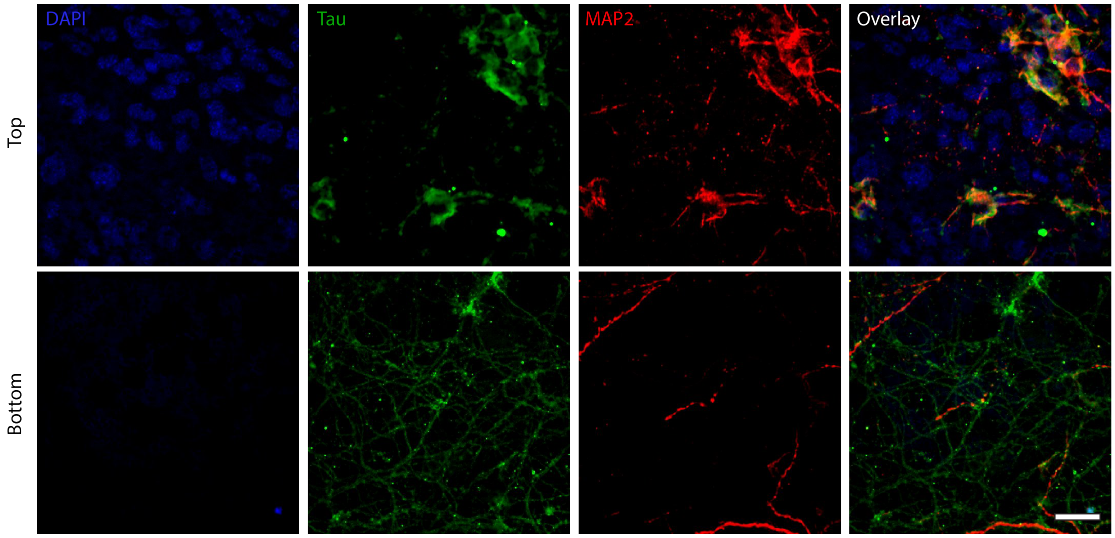

Supplementary Figure 4

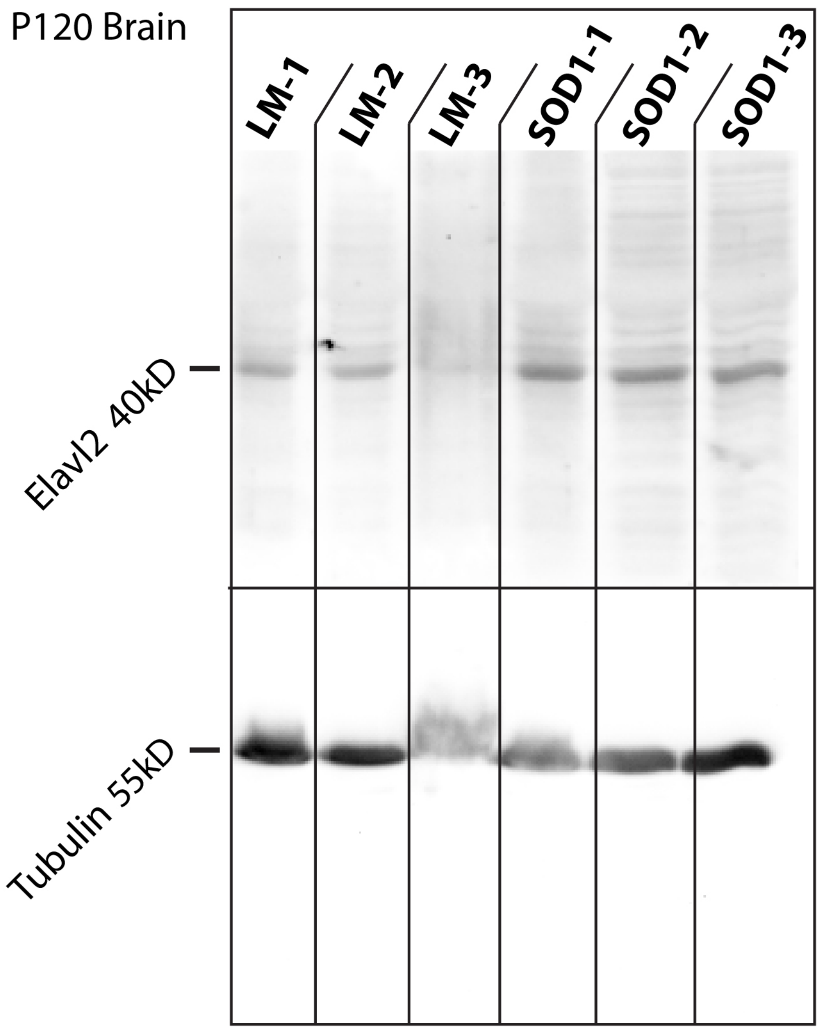

Supplement: Supplementary Figures [file srep44500-s1.pdf]
